# A novel meningococcal outer membrane vesicle vaccine with constitutive expression of FetA: A phase I clinical trial

**DOI:** 10.1016/j.jinf.2015.05.006

**Published:** 2015-09

**Authors:** L. Marsay, C. Dold, C.A. Green, C.S. Rollier, G. Norheim, M. Sadarangani, M. Shanyinde, C. Brehony, A.J. Thompson, H. Sanders, H. Chan, K. Haworth, J.P. Derrick, I.M. Feavers, M.C. Maiden, A.J. Pollard

**Affiliations:** a Oxford Vaccine Group, Department of Paediatrics, University of Oxford and the NIHR Oxford Biomedical Research Centre, CCVTM, Churchill Lane, Oxford OX37LE, United Kingdom; b Nuffield Department of Primary Health Care Sciences, Primary Care Clinical Trials Unit, University of Oxford, 23-38 Hythe Bridge Street, Oxford, United Kingdom; c Department of Zoology, University of Oxford, South Parks Road, United Kingdom; d National Institute for Biological Standards and Control, Blanche Lane, South Mimms, Potters Bar, Hertfordshire, United Kingdom; e Michael Smith Building, Faculty of Life Sciences, University of Manchester, Manchester, United Kingdom

**Keywords:** *Neisseria meningitidis*, Vaccine, Outer membrane vesicles, FetA, Molecular epidemiology

## Abstract

**Objectives:**

Outer membrane vesicle (OMV) vaccines are used against outbreaks of capsular group B *Neisseria meningitidis* (MenB) caused by strains expressing particular PorA outer membrane proteins (OMPs). Ferric enterobactin receptor (FetA) is another variable OMP that induces type-specific bactericidal antibodies, and the combination of judiciously chosen PorA and FetA variants in vaccine formulations is a potential approach to broaden protection of such vaccines.

**Methods:**

The OMV vaccine MenPF-1 was generated by genetically modifying *N. meningitidis* strain 44/76 to constitutively express FetA. Three doses of 25 μg or 50 μg of MenPF-1 were delivered intra-muscularly to 52 healthy adults.

**Results:**

MenPF-1 was safe and well tolerated. Immunogenicity was measured by serum bactericidal assay (SBA) against wild-type and isogenic mutant strains. After 3 doses, the proportion of volunteers with SBA titres ≥1:4 (the putative protective titre) was 98% for the wild-type strain, and 77% for the strain 44/76 FetA_*on*_PorA_*off*_ compared to 51% in the strain 44/76 FetA_*off*_PorA_*off*_, demonstrating that vaccination with MenPF-1 simultaneously induced FetA and PorA bactericidal antibodies.

**Conclusion:**

This study provides a proof-of-concept for generating bactericidal antibodies against FetA after OMV vaccination in humans. Prevalence-based choice of PorA and FetA types can be used to formulate a vaccine for broad protection against MenB disease.

## Highlights

• MenB OMV vaccines' efficacy is strain-restricted by the variable antigen PorA.
• FetA is another variable antigen, but has iron-dependent expression.
• The combination of only a few PorA and FetA can induce broad-protection.
• A mutated OMV was created containing one PorA and one FetA.
• FetA induces bactericidal antibody response in addition to the PorA response in a Phase I trial.

## Introduction

Capsular group B *Neisseria meningitidis* (MenB) is the predominant cause of invasive meningococcal disease (IMD) in most European countries.^1^ In the USA, groups B, C and Y cause IMD in similar proportions.^2^ Invasive MenB disease has declined recently, but still caused 595/769 (77%) of all cases of IMD in the UK in 2012/13,^3^ with an incidence of 1.8/100,000 per year in the period 2006–2012.^4^ The rapid onset of severe disease, potentially resulting in death or significant disability, maintains MenB as a priority for vaccine development. Successes with meningococcal A, C,Y and W polysaccharide–protein conjugate vaccines have not been reproduced with MenB, perhaps due to antigenic similarity of group B capsule sialic acid and human foetal neuronal cells, which is also a safety concern.^5,6^ This has led to the development of formulations based on outer membrane vesicles (OMVs) containing PorA and other outer membrane proteins.^7^ OMV vaccines have been shown to be safe, to induce protective serosubtype (PorA)-specific immune responses, and have been used to successfully control clonal outbreaks of MenB disease.^8–14^ However, these vaccines offer limited protection against different serosubtypes (PorA types) of MenB. The vaccine 4CMenB, recently licensed in Australia, Europe, Canada and the US, includes an OMV component in addition to recombinant proteins^15^ to induce protection against one PorA type.^16^

The PorA type-specificity of OMV vaccines occurs because most bactericidal antibodies are directed against specific surface-exposed epitopes on PorA, which are highly variable. This means that multiple PorA types are required to cover different strains.^17–19^ FetA is an additional vaccine candidate, being a variable subcapsular antigen that has been shown to induce bactericidal antibodies in animal models^20–23^ and to a certain extent during clinical trials, where immune responses against FetA can be detected.^21,24–26^ As a consequence of iron-dependent regulation of its expression during culture,^27^ the amount of FetA is variable in OMV vaccines, ranging from 0 to 9% of the total protein content of MenBVac and MeNZB, used in Norway and New Zealand, respectively.^28,29^ FetA is a TonB-coupled transporter, but its physiologically relevant substrate is unclear: FetA from *N. meningitidis* has been shown to bind ferric iron *in vitro*.^30^ Studies on FetA from *Neisseria gonorrhoeae* have shown that it can transport a range of ferric catecholate-type siderophores.^31^

Hyper-invasive lineages, those meningococcal genotypes causing the majority of invasive disease, exhibit stable, non-overlapping structures of their variable antigens, which limits antigenic diversity.^32^ Analysis of the molecular epidemiology of 4057 clinical IMD isolates obtained between 2000 and 2002, across 18 European countries, revealed that 5/31 clonal complexes accounted for 77% of isolates.^33,34^ Eight out of 273 PorA types accounted for 60% of isolates, and 6/99 FetA types accounted for 67% of isolates (Unpublished results). Therefore, although these antigens are diverse, only a few combinations of subtypes are responsible for the majority of IMD and choice of a limited number of PorA and FetA protein combinations based on surveillance data has potential in designing a vaccine that harnesses the immunogenicity of these proteins. As FetA immunogenicity is less certain than PorA, which is immunodominant, we aimed to demonstrate that constitutive expression of FetA in an OMV vaccine simultaneously induces FetA and PorA bactericidal responses, to provide a proof-of-concept for a PorA–FetA vaccine. Such a vaccine could contain a combination of several proteins that covers the majority of global meningococcal disease. An OMV vaccine expressing a defined and consistent quantity of FetA was produced to express a single PorA and a single FetA (MenPF-1). This novel vaccine was tested in a phase I clinical trial to examine safety, tolerability and immunogenicity in healthy adults.

## Materials and methods

### Molecular epidemiology and estimation of hypothetical vaccine coverage

A vaccine recipe based on that from Russell et al.^35^ was used to estimate potential coverage of a PorA/FetA vaccine based on meningococcal disease isolates collected over a number of decades in England and Wales, which had been characterised by PorA and FetA variable region (VR) sequence typing. This included the 323 disease isolates from 1975, 1985 and 1995,^35^ 150 disease isolates from England and Wales from the EUMenNet study^34^ (http://pubmlst.org/) and 1381 disease isolates from the Meningitis Research Foundation Meningococcus Genome Library (MRF MGL) (http://www.meningitis.org/research/genome). The EUMenNet isolates were from the years 2000–2002 inclusive and the MRF genome library ones were from the epidemiological years 07/2010–06/2011 to 07/2012–06/2013 inclusive. Isolates with an exact match to at least one of the five PorA VR1, VR2 and FetA VRs were considered to be potentially covered by the vaccine. The vaccine components were: VR1: 5-1, 5, 7-2, 7, 19; VR2: 2-2, 2, 4, 15, 10; FetA VR: F1-5, F3-6, F5-1, F3-9, F4-1. Estimated incidence of meningococcal disease calculated from: 1912–1997 notification data; 1998–2011 laboratory reports, 2002 EU-IBIS report,^36^ 2012 Health Protection Report^37^ and Gray et al., 2006.^38^ Reporting criteria and methods have changed on multiple occasions. England and Wales population data was obtained from the Office for National Statistics (ONS) (http://www.ons.gov.uk/).

### Ethics and approvals

Clinical Trial Authorisation was granted by the United Kingdom Medicines and Healthcare products Regulatory Agency (MHRA reference 21584/0298/001-0001). Ethical approval and amendments were granted by NRES Oxford A (12/SC/0023). The trial was registered with clinicaltrials.gov and EudraCT (NCT01640652 and 2012-001046-17 respectively). The trial was conducted in accordance with the clinical trial protocol and the principles of the Declaration of Helsinki (2008) and the International Conference on Harmonization (ICH) Good Clinical Practices standards.

### Vaccine construction and preparation

MenPF-1 was produced to Good Manufacturing Practice (GMP) standard at the Norwegian Institute of Public Health (NIPH) from a genetically modified 44/76 *N. meningitidis* strain (PorA variant P1.7,16 and FetA variant F3-3), as described for the Norwegian OMV vaccine.^29,39^ The wild-type promoter region of the *fetA* gene was replaced with a 17 bp spacer region derived from the promoter region of *porA*, removing iron regulation of FetA expression and ablating slipped strand mis-pairing; the result was a genetically modified 44/76 strain with constitutive expression of FetA and wild-type expression of PorA (unpublished data). OMVs were obtained by detergent extraction, the vaccine consisted of OMVs in sterile solution formulated with aluminium hydroxide, 3% sucrose and water (unpublished data). The vaccine contained 21.8% PorA and 7.7% FetA (of total protein). To investigate whether the FetA levels were abnormally high in the vaccine strain, making the strain more susceptible to killing in the SBA assay, relative to the natural isolate, QC data on the vaccine were compared with existing data on iron-induced FetA expression in the wild-type strain. Frasch et al. reported that wild-type 44/76 OMVs contain 0–10% FetA.^29^ The FetA content of dOMVs from the mutant strain was 7.7%, so the expression in the mutant is within the capabilities of the wild-type strain under certain growth conditions. PorA (21.8% protein) is also within range of the wild-type. Vaccination was with 0.5 ml vaccine for the 25 μg dose or 1.0 ml for the 50 μg dose.

### Phase I study design

The study was an open-label, dose-escalation, single-site, phase I clinical trial in healthy adults. Three doses of 25 μg or 50 μg MenPF-1 were given intra-muscularly 8 weeks apart. Twenty six volunteers were assigned to each dosing group by sequential allocation, and invited to attend 18 visits over 20 weeks (at 0 h, 4–6 h, 24 h, 7, 14 and 28 days after each vaccination).

### Study participants and eligibility criteria

Male and female (using contraception) participants who fulfilled all of the inclusion criteria were enrolled into the trial. Inclusion criteria were ability to give informed consent, aged between 18 and 50 years, in good health, able to attend visits, willing to allow GP/consultant to be notified of their involvement in the study and confirmation from their GP that they were suitable for inclusion in the trial. All volunteers provided informed consent in writing prior to any study procedures. Volunteers were excluded if they had any history of significant organ or system disease, known or suspected alteration in immune function (including IgA deficiency and autoimmune disease), previous receipt of a MenB vaccine of any kind, previous disease caused by *N. meningitidis* or any other significant disease or disorder that presented potential for risk, could influence the results or impair the participants ability to participate in the study.

### Safety monitoring

Frequency and severity of solicited and unsolicited local and systemic adverse events were monitored and reported by study participants for one week after vaccination using electronic diary cards. Adverse events were graded using modified Food and Drug Administration (FDA) and Division of AIDS (DAIDs) criteria (Tables S1 and S2). Volunteers were observed for 1 h post vaccination for immediate adverse reactions. Full blood counts and differential serum renal and liver biochemistry, C-reactive protein and amylase and visit observations (pulse, respiratory rate, blood pressure) were recorded at all visits. An independent data safety monitoring committee provided real-time safety oversight for the duration of the trial, approved safety interpretation and gave formal approval for dose-escalation.

### Analysis of bactericidal responses by Serum Bactericidal Assay (SBA)

Functional antibody responses were measured using SBA with human complement, as previously described.^40^ The SBA was performed on sera from all volunteers available at baseline and after the first or second dose against several bacterial target strains. The isogenic mutant 44/76 FetA_*on*_PorA_*on*_ (SMenPF1.2) was the strain used to produce the OMVs in the MenPF-1 vaccine, and the strains 44/76 FetA_*off*_PorA_*off*_, 44/76 FetA_*off*_PorA_*on*_ and 44/76 FetA_*on*_PorA_*off*_ were created to assess the independent contributions of PorA and FetA antibodies (Table 1). The level of expression of PorA and FetA were measured on the modified strains by SDS-PAGE, as SBA responses are likely to be affected by expression levels.^41^ In the 2 strains constitutively expressing FetA (FetA_*on*_), there was similar amount of FetA, irrespective of whether PorA was on or off. Similarly, there was a similar amount of PorA in the mutant strains expressing or not FetA. There were also similar amounts of other components in all 4 mutant strains. Exogenous human complement without intrinsic bactericidal activity was sourced from consenting healthy adults and used at 25% (vol/vol) with each target strain having a single complement source. Sera obtained from clotted blood samples, spun for 10 min, were stored at −80 °C prior to heat inactivation at 56 °C for 30 min. Bacterial strains were grown overnight on blood agar plates at 37 °C and 5% CO_2_. Approximately 50 colonies were sub-cultured for 4 h, reconstituted in Hanks buffered salt solution (Gibco) with 0.5% bovine serum albumin (Sigma Aldrich). The bacteria were diluted to give approximately 100 colony forming units per 10 μl used for the assay. The SBA titre was defined as the reciprocal of the highest dilution of serum that yielded ≥50% decrease in colony forming units relative to that of control wells within 60 min at 37 °C.

### Statistics

The sample size of each cohort is comparable to studies of other capsular group B OMV vaccines that assessed the safety and tolerability of three doses in healthy adult volunteers.^42^ All available data were used and presented according to the assigned dose group. Analyses for the primary endpoints were descriptively summarised. Severity and number of days for each adverse systemic or local reaction was presented as counts and proportions or percentages per dose groups, with 95% CI calculated using the binomial exact method. Missing e-diary reported data were assumed to correspond to no symptoms on that day. No formal hypothesis tests were carried out. Immunological data were, where appropriate, log_10_-transformed prior to analyses, and back-transformed to be presented as geometric means with 95% confidence intervals according to each time point and group. Missing data due to laboratory processing issues were considered as missing completely at random. Statistical analyses were performed using STATA version 13.1 (StataCorp LP, Texas, USA) and SAS version 9.3 (SAS Institute, Cary, NC, USA).

## Results

### Molecular epidemiology-based design of the vaccine

**Figure 1:**
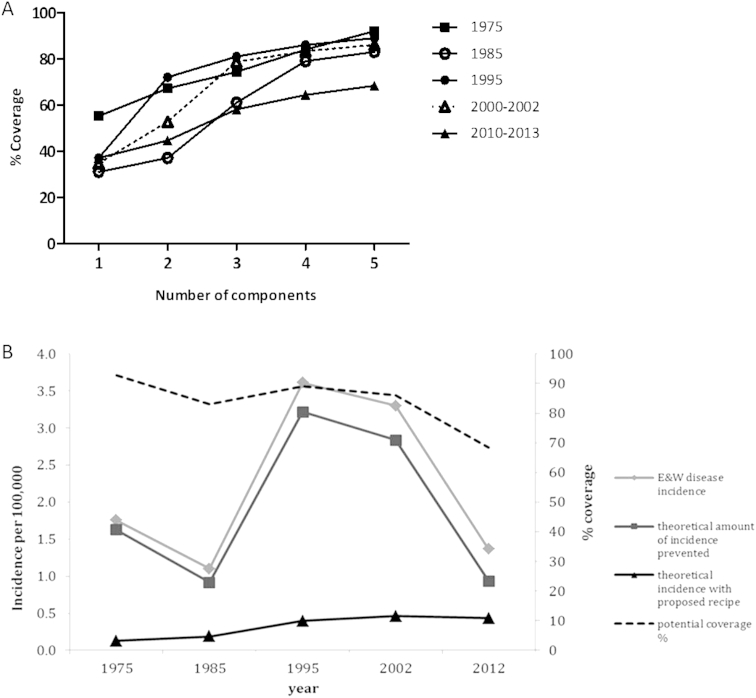
**A vaccine recipe (5PorA–FetA) based on that from Russell et al., 2008****^35^****was used to estimate potential coverage and longevity of a PorA/FetA based recipe over a number of decades in England and Wales. (A)** Potential coverage of PorA/FetA recipes^35^ in England and Wales 1975–1995, 2000–2002 and 2010–2013 dataset with increasing number of components (PorA variable regions – VRs). **(B)** Theoretical amount of disease incidence prevented/present if 5PorA/FetA recipe was implemented in England and Wales 1975–2012. Isolates with an exact match to at least one of the five PorA VR1, VR2 and FetA VRs were considered to be covered by the recipe.

A hypothetical vaccine containing a combination of five PorA and five FetA proteins (5PorA–FetA) (Table 2) could in principle have conferred protection against 83–93% of cases of IMD in England and Wales between the period 1975 and 2002 (Fig. 1A). After 2002, potential coverage of this 5PorA–FetA recipe reduced to 68%, which is a consequence of natural fluctuations in meningococcal types. We modelled an impact of the 5PorA–FetA vaccine using incidence data from between 1975 and 2012 (Fig. 1B) and found that a 5-valent vaccine with 100% efficacy would have reduced invasive disease incidence to below 0.5/100,000 across this period. This compares with the actual incidence in 1985 and 1995 of 1.10 and 3.61/100,000 respectively. This retrospective analysis demonstrates the potential impact of PorA and FetA multicomponent vaccines on disease rates in recent decades. This vaccine design is based on the contention that a vaccine containing both PorA and FetA antigens induces simultaneous FetA and PorA bactericidal responses in humans. To test this, an OMV vaccine expressing a defined and consistent quantity of FetA was produced to express a single PorA and a single FetA (MenPF-1). This novel vaccine was tested in a phase I clinical trial to examine safety, tolerability and immunogenicity in healthy adults.

### Phase I trial recruitment and population

**Figure 2:**
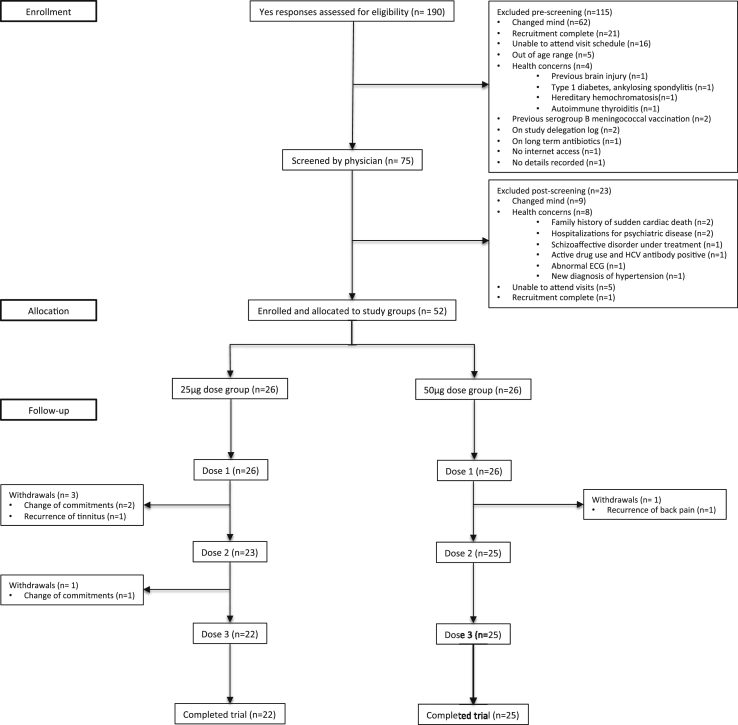
**Flow diagram for recruitment, enrolment and completion of trial**. Withdrawals were not related to the study vaccine and all new and medically relevant information that was detected at screening were communicated to the candidate volunteers' General Practitioners with their permission.

Recruitment commenced in October 2012; 75 volunteers were assessed by the trial physicians and 52 were enrolled. The final clinical trial data were collected one month after the last vaccine dose (June 2013). Recruitment, enrolment and withdrawals are presented in Fig. 2. Overall, 856/872 (98.2%) of all attended visits were performed within the protocol-defined window after vaccination. The baseline physical and demographic characteristics of enrolled volunteers are presented in Table 3.

### Safety of MenPF-1 in healthy adults

There were no serious adverse events, serious adverse reactions, severe unexpected serious adverse reactions or medically significant adverse events. Adverse events to vaccination were mostly local reactions at the site of injection, mild to moderate in severity and transient self-limiting reactions that lasted only a few days (Table 4). There was no evidence of cumulative adverse event frequency or severity with repeated doses of vaccine and systemic symptoms such as fever were infrequent (Table 4). Visit observations and safety blood testing did not show any safety concerns (data not shown).

### Serum bactericidal antibody responses are induced against homologous wild-type strain 44/76

**Figure 3:**
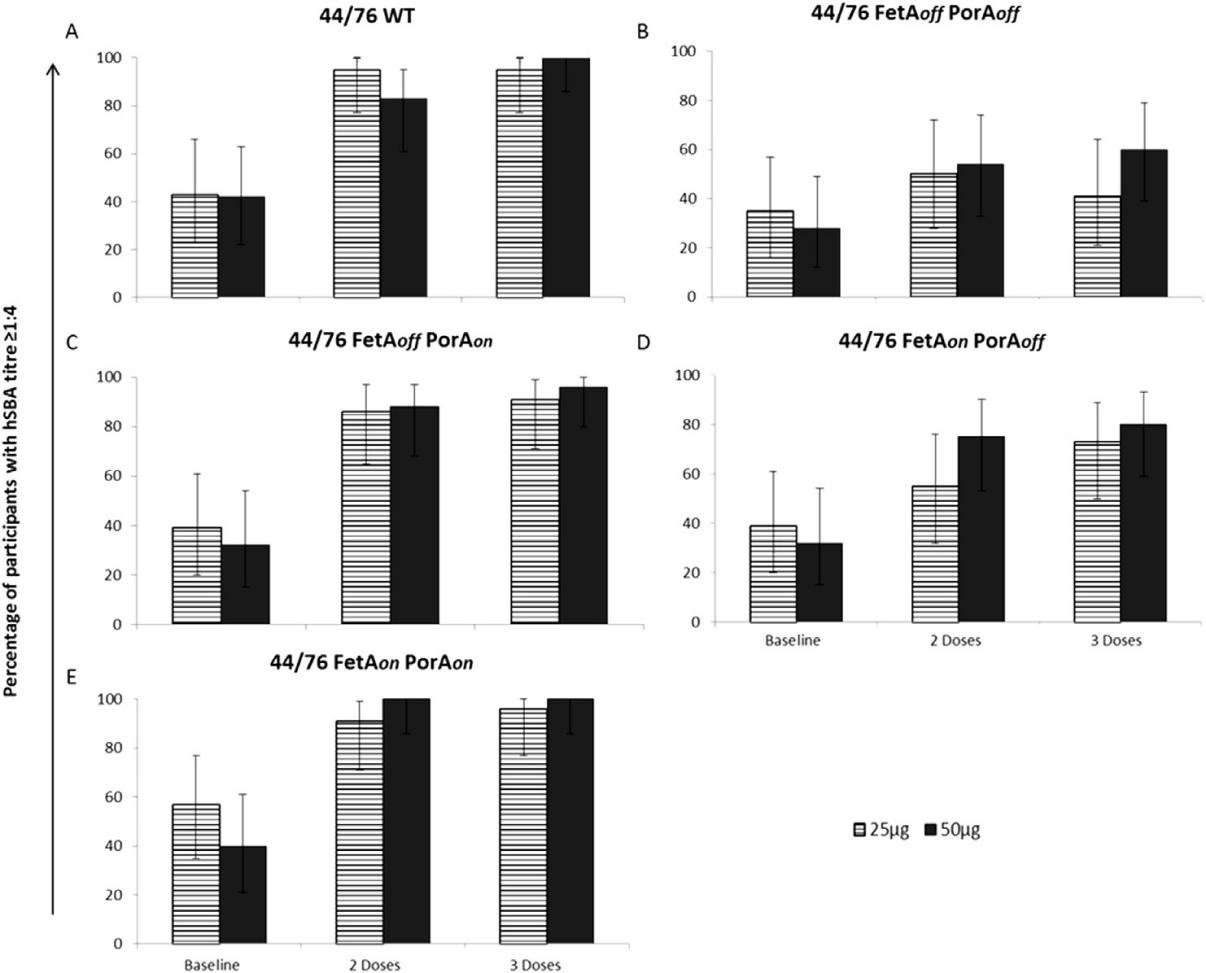
**Percentage of participants that had an SBA titre ≥1:4 at baseline and 4 weeks after 2 or 3 doses of MenPF-1**. Percentage of participants with an SBA titre ≥1:4 as main bars (with 95% CI error bars) for participants receiving 25 μg of vaccine (lines) and 50 μg of vaccine (dark solid) at baseline and 4 weeks after 2 or 3 doses of MenPF-1. SBA titres were determined as the reciprocal dilution where bacterial survival was less than 50% of that of controls for the parental wild-type strain 44/76 **(A)**, 44/76 FetA_*off*_PorA_*off*_**(B)**, 44/76 FetA_*off*_PorA_*on*_**(C)**, 44/76 FetA_*on*_PorA_*off*_**(D)** and 44/76 FetA_*on*_PorA_*on*_**(E)**.

Baseline sera were available for all participants, 46/52 at four weeks after the second dose and 47/52 at four weeks post third dose. The majority of volunteers developed an SBA titre ≥1:4 against the homologous 44/76 WT strain after 3 doses in both the 25 μg (95%) and 50 μg dose groups (100%) (Fig. 3A), as compared to 42% and 43%, respectively, at baseline. The baseline geometric mean titres (GMTs) were 4.8 and 3.5 in participants receiving 25 μg and 50 μg doses respectively, indicating low levels of naturally acquired SBA activity in most volunteers prior to vaccination. After 3 doses of MenPF-1 the GMTs increased to 24.0 and 31.1 in the low and high dose groups respectively (Table 5). The study was not powered to investigate whether there was significant differences between 2 or 3 doses or between the 25 or 50 μg doses.

### MenPF-1 induced PorA-specific serum bactericidal antibodies as expected

Isogenic target strains were used to investigate the relative contributions of FetA and PorA antibodies towards bactericidal activity following MenPF-1 vaccination. The proportion of volunteers with baseline SBA activity ≥1:4 against the target strain 44/76 FetA_*off*_PorA_*off*_ were 35% and 28% in the 25 μg and 50 μg dose groups respectively, increasing to 41% and 60% respectively after 3 doses of MenPF-1 (Fig. 3B). The proportion of responders to the isogenic strain 44/76 FetA_*off*_PorA_*on*_ were 39% and 32% in the 25 μg and 50 μg dose groups respectively at baseline, increasing to 91% and 96% after 3 doses (Fig. 3C). The GMT values after 3 doses against the strain 44/76 FetA_*off*_PorA_*off*_ were 3.6 (95% CI 2.5, 5.4) and 6.6 (95% CI 3.6, 11.9) in the 25 μg and 50 μg dose groups respectively, as compared to 19.9 (95% CI 9.9, 40.1) and 29.4 (95% CI 16.4, 52.9) against 44/76 FetA_*off*_PorA_*on*_ (Table 5). These results demonstrate that a PorA-specific bactericidal response was induced in most participants.

### MenPF-1 induced FetA-specific serum bactericidal antibodies

Most importantly, the isogenic strain 44/76 FetA_*on*_PorA_*off*_ was designed to quantify the FetA-specific bactericidal response. The proportion of responders with SBA activity ≥1:4 against this strain were 39% (25 μg dose) and 32% (50 μg) at baseline compared to 73% and 80% after 3 doses of MenPF-1 (Fig. 3D). The proportion of responders was greater against 44/76 FetA_*on*_PorA_*off*_ than was observed against strain 44/76 FetA_*off*_PorA_*off*_, which lacks expression of FetA (Fig. 3B). The GMTs were also greater against 44/76 FetA_*on*_PorA_*off*_ than 44/76 FetA_*off*_PorA_*off*_ in both the 25 μg and 50 μg dose groups after 3 doses, with values of 7.3 (95% CI 4.6, 11.6) and 11.2 (95% CI 6.5, 19.2) respectively, vs. 3.6 (95% CI 2.5, 5.4) and 6.6 (95% CI 3.6, 11.9). These results indicate that MenPF-1 was able to induce FetA-specific bactericidal antibodies.

In addition, when 44/76 FetA_*on*_PorA_*on*_ (vaccine strain) was the target strain for SBA, the proportion of baseline responders with titres ≥1:4 was 57% in the 25 μg dose group and 40% in the 50 μg dose group (Fig. 3E), increasing to 96% and 100% after 3 doses of MenPF-1. In both the 25 μg and 50 μg dose groups the GMTs against 44/76 FetA_*on*_PorA_*on*_ were higher than those detected against the FetA_*off*_PorA_*on*_ strain, where FetA expression is absent. The GMTs in the high dose group were 4.2 (95% CI 2.8, 6.4), 42.7 (95% CI 23.1, 79.1) and 49.9 (95% CI 29.8, 83.5) at baseline, after 2 and 3 doses respectively, as compared to the FetA_*off*_PorA_*on*_ strain where GMTs of 3.0 (95% CI 2.3, 4.0), 26.1 (95% CI 12.8, 53.4) and 29.4 (95% CI 16.4, 52.9) were observed respectively. This result suggests that FetA-specific bactericidal antibodies generated in response to MenPF-1 were additive to the PorA-specific SBA responses.

## Discussion

We demonstrated that immunisation with OMVs derived from a meningococcal strain that constitutively expressed FetA elicited both PorA and FetA-specific bactericidal antibodies in humans, providing evidence that a multicomponent vaccine based on PorA and FetA could be used to provide protection against meningococcal disease. In order to assess the contribution of FetA bactericidal antibodies, SBA assays were conducted targeting isogenic strains in which the level of FetA expression was measured.^41^ Immunisation with MenPF-1 led to elevated GMTs in SBA against the target strain 44/76 FetA_*on*_PorA_*on*_ in comparison to wild-type 44/76. Furthermore, the bactericidal activity against 44/76 FetA_*on*_PorA_*off*_ indicated a FetA-specific response, which was greater at the last time-point, illustrating the benefit of the third dose. While immune responses against FetA have been observed in convalescent patients after meningococcal disease,^43^ the present trial demonstrates that such bactericidal activity can be induced against the antigen through vaccination with OMVs.

In addition to being immunogenic in healthy adults MenPF1 was safe. The greater reporting of pain and tenderness at the site of injection from volunteers who received 50 μg may be a true dose effect or a confounding observation due to doubling the vaccine volume (to 1 ml) and/or doubling the quantity of adjuvant, which was necessary in this study for dose escalation. Missing solicited symptom data were assumed to correspond to no symptoms on that day; however, these missing data accounted for only 634/10,291 (6.2%) of solicited symptom data points. Overall the side effects reported in this trial are comparable to those observed following use of other OMV MenB vaccines in healthy adults.^7^ The low frequency of fever is in contrast to infant fever previously observed with 25 μg dose OMV vaccines^44^ possibly due to an age-specific effect. Adverse events were also reported in adults following vaccination with 4CMenB, a multicomponent MenB vaccine containing the same OMV as MeNZB,^45^ although this is not directly comparable because it also contains three recombinant proteins, which may contribute to reactogenicity.

We have confirmed the immunogenicity of the MenPF-1 formulation against the homologous vaccine strain as well as the wild-type and isogenic strains. While it is known that SBA titres tend to differ amongst laboratories,^46^ the titres against 44/76 in adults immunised with three 25 μg doses of MenBVac were comparable to those recorded for the low dose group in the present study^46^ and the titres seen after 3 50 μg doses of MenPF-1 were similar to those seen against strain NZ98/294 in adults vaccinated with 3 doses of 4CMenB.^47^ As was seen for MenBVac,^46^ participants immunised with MenPF-1 demonstrated an increase in functional antibodies against 44/76 after the second and third doses. A considerable proportion of the increase in functional antibodies was directed against PorA as demonstrated by the lower GMTs detected against the isogenic strains with PorA expression removed. The induction of PorA-specific functional responses is in agreement with previous capsular group B OMV vaccine trials.^48^ As an immunodominant antigen in the meningococcus, PorA responses account for a large proportion of detected bactericidal antibodies against homologous target strains. Coupled with this, P1.7,16 has been consistently shown to be more immunogenic than other PorA variants^48–50^ and was expected to play a major role in inducing bactericidal activity in this study. The relative immunogenicity of different FetA types have not yet been determined and could also be variable.

The baseline levels of anti-FetA and PorA bactericidal activity are probably attributable to prior natural exposure to circulating strains of *N. meningitidis*. Meningococci of the ST-32 complex were found in 6.5% of IMD isolates in England and Wales between 2006 and 2009.^51^ The PorA–FetA combination in the current study is common amongst the ST-32 complex organisms.^52^ While not characterised in the current study, there are a number of other antigens that may have contributed to the immunogenicity of MenPF-1. Relatively high pre-vaccination levels of SBA against 44/76 FetA_*off*_PorA_*off*_ demonstrate that a considerable proportion of subjects had functional antibodies against a number of non-PorA or FetA antigens, which increased after MenPF-1 vaccination. Some minor antigens have been identified previously using OMV vaccines including TbpA, Hsf and NspA.^53^

While both PorA and FetA-specific bactericidal responses were induced, the latter was possibly limited by the expression level of FetA (around 7%), in comparison to PorA (21.8%). Therefore, given the potential for inducing FetA specific antibodies, future improvements would include enhanced expression of FetA. Furthermore, OMV vaccine-induced bactericidal antibodies to PorA variant P1.7,16 show very limited cross-reactivity against heterologous variants,^19,48^ thus this single-strain OMV vaccine would not confer broad protection against diverse MenB disease. Therefore, future studies will focus on combining and evaluating the response to PorA and FetA variants chosen to broaden the coverage of epidemiologically relevant MenB strains. As PorA and FetA data are used as part of the routine surveillance of meningococcal disease across many reference labs, changes associated with IMD could be used to inform PorA/FetA vaccine combinations to maintain optimal coverage. In addition, such a vaccine would not be based on the bacterial capsule, so could potentially protect against meningococci expressing other capsular groups.

## Funding

The trial was funded by a Wellcome Trust Strategic Translation Award (Award number: 082102/Z/07/A) and supported by the Oxford Partnership Comprehensive Biomedical Research Centre with funding from the Department of Health's National Institute of Health Research, Biomedical Research Centres funding scheme. The trial was performed by the University of Oxford at the Centre for Clinical Vaccinology and Tropical Medicine, Oxford, and monitored by the Clinical Trial Research Governance department, University of Oxford. CD, LM and CR were partly supported by grants from Meningitis UK to CR, and from Action Medical Research (SP4594) to AJP. CR is a Jenner Institute investigator and an Oxford Martin fellow.

## Author contributions

CAG was the lead physician throughout the trial, wrote the protocol with GN, CR and MS and prepared regulatory submissions, screened and recruited volunteers, managed the clinical part of the trial, collated and analysed the primary endpoint data. KH was the lead nurse on the study. CAG, LM and CD wrote the manuscript in collaboration with CB. CR and AJT managed the laboratory aspect of the trial (lab analysis plan, sample receipt, processing, and archiving). LM and CD optimised, validated and performed all the SBA analyses. MS performed the tabulations and statistical analyses in accordance to the Statistical Analysis Plan and trial protocol. AJT, CR, LM and CD processed laboratory samples. HS constructed the homologous and isogenic strains used in the SBA and GN was responsible for the GMP production of MenPF-1 at NIPH. CB and MM performed the epidemiological analysis to inform vaccine antigen selection. GN, MS, JD, IF, CR, MM and AJP instigated this research and designed the clinical trial. All authors had input into the manuscript and approved the manuscript for publication.

## Competing interests

AJP has previously conducted clinical trials on behalf of Oxford University funded by manufacturers of meningococcal vaccines including Novartis Vaccines, GlaxoSmithKline, Pfizer and Sanofi Pasteur. He does not receive any personal payments from them nor travel reimbursement or honoraria. His department has received unrestricted educational grants from vaccine manufacturers for organisation of courses and symposia. AJP has previously been named on patents in the field of group B meningococcal vaccines and is Chair of the UK Joint Committee on Vaccines and Immunisation and the European Medicine Agency's Scientific Advisory Group on Vaccines. MS is a co-investigator on investigator-initiated research grants from Pfizer. MM undertakes occasional consultancy work for Pfizer, GSK and Novartis. IF and HC are employees at NIBSC, a centre of the Medicines and Healthcare products Regulatory Agency. The clinical trial was approved by the MHRA prior to the merger of NIBSC with the agency. CG, LM, CR, JD, CD, HS, KH, GN and CB no conflict. The views expressed in this publication are those of the authors and not necessarily those of the Department of Health.

## Figures and Tables

**Table 1:** Bacterial strains used in the Serum Bactericidal Assay.

Strain	Genotype	Study name	PorA expression	FetA expression
WT	44/76 (wild-type)	44/76 WT	On	Iron restricted
SMenPF1.2	44/76 *fetAp*_*17bp*_	44/76 FetA_*on*_PorA_*on*_	On	On
3043	44/76 *fetA::kan*	44/76 FetA_*off*_PorA_*on*_	On	Off
3311	44/76 *fetA::kan porA::ery*	44/76 FetA_*off*_PorA_*off*_	Off	Off
3312	44/76 *fetAp*_*17bp*_*porA::ery*	44/76 FetA_*on*_PorA_*off*_	Off	On

(WT) 44/76 (wild-type) with constitutive PorA expression and iron-dependent FetA expression. (SMenPF1.2) 44/76 *fetAp*_*17bp*_ is derived from the MenPF-1 GMO strain and constitutively expresses both PorA and FetA. (3043) 44/76 *fetA::kan* expression of FetA is interrupted by kanamycin resistance gene insertion. (3311) 44/76 *fetA::kan porA::ery* kanamycin and erythromycin resistance gene insertions used to interrupt FetA and PorA expression respectively. (3312) 44/76 *fetAp*_*17bp*_*porA::ery* constitutively expresses FetA with PorA expression interrupted by insertion of erythromycin resistance gene.

**Table 2:** Components of Russell et al. PorA/FetA recipe in Fig. 1.

Recipe	FetA VR	VR1	VR2
1 component	F1-5	5-1	2-2

2 components	F1-5	5-1	2-2
	F3-6	5	2

3 components	F1-5	5-1	2-2
	F3-6	5	2
	F5-1	7-2	4

4 components	F1-5	5-1	2-2
	F3-6	5	2
	F5-1	7-2	4
	F3-9	7	15

5 components^a^	F1-5	5-1	2-2
	F3-6	5	2
	F5-1	7-2	4
	F3-9	7	15
	F4-1	19	10

a The 5PorA/FetA vaccine recipe used to determine theoretical amount of disease incidence prevented/present if PorA/FetA vaccine recipe was applied to meningococcal disease incidence in England and Wales 1975–2012.

**Table 3:** Baseline physical and demographic characteristics of study volunteers enrolled.

		25 μg dose group	50 μg dose group
N enrolled	Dose 1	26	26
	Dose 2	23	25
	Dose 3	22	25
Median age at enrolment, years (min–max)		30 (19–49)	24 (18–44)
Male (%)		17 (65.4)	10 (38.5)
Ethnicity by count	White British	23	16
	White (Other)	1	7
	White (Irish)	0	1
	Pakistani	1	0
	Chinese	1	0
	Black (Other)	0	1
	Mixed	0	1

**Table 4:** Frequency of volunteer reported solicited adverse events within one week of each dose of vaccine, presented by severity grade.

Solicited symptoms within one week		Dose 1					Dose 2					Dose 3				
		n	Any (%)	Mild (%)	Mod (%)	Sev (%)	n	Any (%)	Mild (%)	Mod (%)	Sev (%)	n	Any (%)	Mild (%)	Mod (%)	Sev (%)
Tenderness/pain at the site of injection	25 μg dose	25	21 (84)	15 (60)	6 (24)	0 (0)	22	21 (95)	17 (77)	3 (14)	1 (5)	21	20 (95)	14 (67)	3 (14)	3 (14)
	50 μg dose	26	25 (96)	10 (39)	13 (50)	2 (8)	23	23 (100)	12 (52)	9 (39)	2 (9)	24	22 (92)	12 (50)	7 (29)	3 13)
Headache	25 μg dose	24	4 (17)	3 (13)	1 (4)	0 (0)	22	9 (41)	6 (27)	3 14)	0 (0)	21	7 (33)	6 (29)	1 (5)	0 (0)
	50 μg dose	26	12 (46)	8 (31)	4 (15)	0 (0)	23	7 (30)	4 (17)	2 (9)	1 (4)	23	7 (30)	3 (13)	3 (13)	1 (4)
Malaise	25 μg dose	24	4 (17)	3 (13)	0 (0)	1 (4)	22	6 (27)	5 (23)	1 (5)	0 (0)	21	5 (24)	2 (10)	2 (10)	1 (5)
	50 μg dose	26	10 (38)	9 (35)	1 (4)	0 (0)	23	7 (30)	3 (13)	4 (17)	0 (0)	23	8 (35)	5 (22)	3 (13)	0 (0)
Myalgia	25 μg dose	24	10 (42)	8 (33)	2 (8)	0 (0)	22	9 (41)	9 (41)	0 (0)	0 (0)	21	11 (52)	7 (33)	3 (14)	1 (5)
	50 μg dose	26	13 (50)	8 (31)	4 (16)	1(4)	23	10 (43)	7 (30)	2 (9)	1 (4)	23	11 (48)	8 (35)	3 (13)	0 (0)
Nausea and/or vomiting	25 μg dose	24	1 (4)	0 (0)	0 (0)	1 (4)	22	1 (5)	1 (5)	0 (0)	0 (0)	21	5 (24)	3 (14)	1 (5)	1 (5)
	50 μg dose	26	2 (8)	2 (8)	0 (0)	0 (0)	23	4 (17)	3 (13)	1 (5)	0 (0)	23	2 (9)	2 (9)	0 (0)	0 (0)
Arthralgia	25 μg dose	24	1 (4)	1 (4)	0 (0)	0 (0)	22	4 (18)	4 (18)	0 (0)	0 (0)	21	4 (19)	3 (15)	1 (5)	0 (0)
	50 μg dose	26	5 (19)	3 (12)	2 (8)	0 (0)	23	3 (13)	2 (9)	0 (0)	1 (4)	23	5 (22)	4 (17)	1 (5)	0 (0)
Oral temperature (fever)	25 μg dose	25	0 (0)	0 (0)	0 (0)	0 (0)	22	1 (5)	0 (0)	1 (5)	0 (0)	21	1 (5)	0 (0)	1 (5)	0 (0)
	50 μg dose	26	0 (0)	0 (0)	0 (0)	0 (0)	23	0 (0)	0 (0)	0 (0)	0 (0)	24	0 (0)	0 (0)	0 (0)	0 (0)
Redness at the site of injection	25 μg dose	25	19 (76)	18 (72)	0 (0)	1 (4)	22	20 (91)	20 (91)	0 (0)	0 (0)	21	19 (90)	17 (81)	0 (0)	2 (10)
	50 μg dose	26	21 (81)	19 (73)	1 (4)	1 (4)	23	21 (91)	18 (78)	0 (0)	3 (13)	24	23 (96)	22 (92)	0 (0)	1 (4)
Induration at the site of injection	25 μg dose	25	11 (44)	11 (44)	0 (0)	0 (0)	22	15 (68)	15 (68)	0 (0)	0 (0)	21	11 (52)	11 (53)	0 (0)	0 (0)
	50 μg dose	26	17 (65)	15 (58)	0 (0)	2 (8)	23	19 (83)	17 (74)	1 (4)	1 (4)	24	13 (54)	12 (50)	0 (0)	1 (4)
Swelling at the site of injection	25 μg dose	25	10 (40)	8 (32)	0 (0)	2 (8)	22	11 (50)	8 (36)	0 (0)	3 (14)	21	11 (52)	9 (43)	1 (5)	1 (5)
	50 μg dose	26	10 (38)	8 (31)	0 (0)	2 (8)	23	16 (70)	13 (57)	0 (0)	3 (13)	24	17 (71)	14 (58)	1 (4)	2 (8)

Volunteers reported symptoms as mild (does not interfere with routine activities), moderate (interferes with routine activities) and severe (unable to perform routine activities). Redness, swelling and induration at the site of injection used the maximal recorded diameter of any reaction for severity grading as mild (1–10 mm), moderate (11–25 mm) and severe (≥26 mm). Oral temperature was graded as mild (38.0–38.4 °C), moderate (38.5–38.9 °C) and severe (≥39.0 °C).

**Table 5:** SBA geometric mean titres at baseline and 4 weeks after 2 or 3 doses of MenPF-1 (95% CI).

Strain	SBA GMTs (95% CI)					
	25 μg dose			50 μg dose		
	Baseline	After 2 doses	After 3 doses	Baseline	After 2 doses	After 3 doses
44/76 WT	4.8 (2.9, 7.9)	17.0 (9.2, 31.5)	24.1 (12.3, 47.3)	3.5 (2.4, 5.0)	23.0 (12.1, 43.8)	31.1 (19.2, 50.5)
44/76 FetA_*on*_PorA_*on*_	6.3 (3.7, 10.7)	21.2 (12.1, 37.4)	30.0 (15.7, 57.5)	2.2 (2.8, 6.4)	42.7 (23.1, 79.1)	49.9 (29.8, 83.5)
44/76 FetA_*off*_PorA_*on*_	4.2 (2.6, 6.8)	14.6 (7.7, 27.6)	19.9 (9.9, 40.1)	3.0 (2.3, 4.0)	26.1 (12.8, 53.4)	29.4 (16.4, 52.9)
44/76 FetA_*off*_PorA_*off*_	3.2 (2.3, 4.6)	4.3 (2.8, 6.4)	3.6 (2.5, 5.4)	2.5 (2.2, 2.9)	4.8 (3.0, 7.8)	6.6 (3.6, 11.9)
44/76 FetA_*on*_PorA_*off*_	4.5 (2.7, 7.6)	4.7 (3.0, 7.3)	7.3 (4.6, 11.6)	3.1 (2.3, 4.3)	7.6 (4.4, 12.9)	11.2 (6.5, 19.2)
