# Supplementary material for: A novel meningococcal outer membrane vesicle vaccine with constitutive expression of FetA: A phase I clinical trial
Source: J Infect. 2015 Sep;71(3):326–37. doi: 10.1016/j.jinf.2015.05.006 (PMC4535279; doi:10.1016/j.jinf.2015.05.006)
Supplement: Supplementary file 1 [file mmc1.docx]

**Supplementary Materials:**

|  | **Grade 1**  **(Mild)** | **Grade 2**  **(Moderate)** | **Grade 3**  **(Severe)** | **Grade 4**  **(Emergency)** |
| --- | --- | --- | --- | --- |
| Oral temperature (̊C) | 38 - 38.4 | 38.5 - 38.9 | >39 | Emergency department visit or hospitalisation |
| Tachycardia (beats per min) | 101-115 | 116-130 | >130 | Emergency department visit or hospitalisation for arrhythmia |
| Bradycardia (beats per min) | 50-54 | 45-49 | <45 | Emergency department visit or hospitalisation for arrhythmia |
| Systolic hyper-tension (mmHg) | 141-150 | 151-155 | >155 | Emergency department visit or hospitalisation |
| Diastolic hyper-tension (mmHg) | 91-95 | 96-100 | >100 | Emergency department visit or hospitalisation |
| Systolic hypo-tension (mmHg) | 85-89 | 80-84 | <80 | Emergency department visit or hospitalisation |
| Respiratory rate (breaths per min) | 17-20 | 21-25 | >25 | Emergency department visit or hospitalisation |

**Supplementary table S1. Modified Food and Drug Administration (FDA) criteria for adverse events grading**.

|  | **Grade 1**  **(Mild)** | **Grade 2**  **(Moderate)** | **Grade 3**  **(Severe)** | **Grade 4**  **(Potentially life threatening)** |
| --- | --- | --- | --- | --- |
| Haemoglobin (female): decrease from baseline value (gm/dl) | < 1.5 | 1.5-2.0 | 2.1-5.0 | >5 |
| Haemoglobin (male): decrease baseline value (gm/dl) | < 1.5 | 1.5-2.0 | 2.1-5.0 | >5 |
| White cell count: elevated (cell/mm^3^) | 10,800–15,000 | 15,001–20,000 | 20,001–25,000 | >25,000 |
| White cell count: depressed (cells/mm^3^) | 2500-3500 | 1500-2499 | 1000-1499 | <1000 |
| Neutrophil count (cells/mm^3^) | 1500-2000 | 1000-1499 | 500-999 | <500 |
| Platelets (cells/mm^3^) | 125,000-140,000 | 100,000-124,000 | 25,000-99,000 | <25,000 |
| Sodium: hyponatraemia (mEq/L) | 132–134 | 130–131 | 125–129 | <125 |
| Sodium: hypernatraemia (mEq/L) | 144–145 | 146–147 | 148–150 | >150 |
| Potassium: hyperkalaemia (mEq/L) | 5.1–5.2 | 5.3–5.4 | 5.5–5.6 | >5.6 |
| Potassium: hypokalaemia (mEq/L) | 3.5–3.6 | 3.3–3.4 | 3.1–3.2 | <3.1 |
| Urea (mmol/L) | 8.2–8.9 | 9.0–11 | >11 | RRT |
| Creatinine (μmol/L) | 132-150 | 151-176 | 177-221 | >221 or RRT |
| ALT and/or AST (IU/L) | 1.1–2.5 x ULN | >2.6–5.0 x ULN | 5.1-10 x ULN | >10 x ULN |
| Bilirubin, with increase in LFTs (umol/L) | 1.1–1.25 x ULN | 1.26–1.5 x ULN | 1.51–1.75 x ULN | >1.75 x ULN |
| Bilirubin, with normal LFTs (umol/L) | 1.1–1.5 x ULN | 1.6–2.0 x ULN | 2.0–3.0 x ULN | >3.0 x ULN |
| Alkaline phosphatase (U/L) | 1.1–2.0 x ULN | 2.1–3.0 x ULN | 3.1–10 x ULN | >10 x ULN |
| Amylase (U/L) | 1.1–1.5 x ULN | 1.6–2.0 x ULN | 2.1–5.0 x ULN | >5.0 x ULN |
| Albumin: hypoalbuminaemia (g/L) | 28–31 | 25–27 | <25 | Not applicable |
| C-reactive protein | >10-30 | 31-100 | 100-200 | >200 |

**Table S2. Modified Division of AIDS (DAIDs) criteria for adverse events grading.**
